# A comparison of the use of adipose-derived and bone marrow-derived stem cells for peripheral nerve regeneration in vitro and in vivo

**DOI:** 10.1186/s13287-020-01661-3

**Published:** 2020-04-09

**Authors:** Li Na Zhou, Jia Chuan Wang, Prince Last Mudenda Zilundu, Ya Qiong Wang, Wen Ping Guo, Sai Xia Zhang, Hui Luo, Jian Hong Zhou, Ru Dong Deng, Dong Feng Chen

**Affiliations:** 1 Department of Anatomy, School of basic medical sciences, Guangzhou University of Chinese Medicine, 232 Waihuan East Road, Guangzhou, 510006 Guangdong China; 2 Department of Pathology, Shenzhen Traditional Chinese Medicine Hospital, The Fourth Clinical Medical College of Guangzhou University of Chinese Medicine, Shenzhen, China; 3 Department of Anatomy, Zhongshan School of Medicine, Sun Yat-Sen University, Guangzhou, China; 4 Department of Electron Microscope, Zhongshan School of Medicine, Sun Yat-Sen University, Guangzhou, China

**Keywords:** Bone marrow-derived stem cells, Adipose-derived stem cells, Schwann cells, Co-culture, Chemically extracted acellular nerve allograft, Peripheral nerve repair

## Abstract

**Background:**

To date, it has repeatedly been demonstrated that infusing bone marrow-derived stem cells (BMSCs) into acellular nerve scaffolds can promote and support axon regeneration through a peripheral nerve defect. However, harvesting BMSCs is an invasive and painful process fraught with a low cellular yield.

**Methods:**

In pursuit of alternative stem cell sources, we isolated stem cells from the inguinal subcutaneous adipose tissue of adult Sprague–Dawley rats (adipose-derived stem cells, ADSCs). We used a co-culture system that allows isolated adult mesenchymal stem cells (MSCs) and Schwann cells (SCs) to grow in the same culture medium but without direct cellular contact. We verified SC phenotype in vitro by cell marker analysis and used red fluorescent protein-tagged ADSCs to detect their fate after being injected into a chemically extracted acellular nerve allograft (CEANA). To compare the regenerative effects of CEANA containing either BMSCs or ADSCs with an autograft and CEANA only on the sciatic nerve defect in vivo, we performed histological and functional assessments up to 16 weeks after grafting.

**Results:**

In vitro, we observed reciprocal beneficial effects of ADSCs and SCs in the ADSC–SC co-culture system. Moreover, ADSCs were able to survive in CEANA for 5 days after in vitro implantation. Sixteen weeks after grafting, all results consistently showed that CEANA infused with BMSCs or ADSCs enhanced injured sciatic nerve repair compared to the acellular CEANA-only treatment. Furthermore, their beneficial effects on sciatic injury regeneration were comparable as histological and functional parameters evaluated showed no statistically significant differences. However, the autograft group was roundly superior to both the BMSC- or ADSC-loaded CEANA groups.

**Conclusion:**

The results of the present study show that ADSCs are a viable alternative stem cell source for treating sciatic nerve injury in lieu of BMSCs.

## Background

Peripheral nerve defects always result in functional loss and remain an intractable challenge for clinical researchers [26, 46, 47, 50]. To mitigate the functional loss, peripheral nerve tissue engineering involving nerve conduits and seeding cells such as Schwann cells (SCs) [17, 45], Schwann-like cells [52], and stem cells [7, 51] are promising alternative interventions. These applications for developing ideal seeding cells are a high priority in peripheral nerve regeneration translational research.

Schwann cells (SCs) are peripheral glial cells that form the myelin coat around the axial cylinder of peripheral nerve fibers [8, 31]. In the event of a nerve injury, SCs and macrophages cooperate to clear distal stump myelin debris in a process known as Wallerian degeneration. They also proliferate greatly to form bands that support axon growth into and through distal nerve stumps. This support includes supplying numerous growth factors and extracellular matrix secretion [13, 23, 25, 27, 33]. Experimentally, SCs transplantation has been shown to enhance axon outgrowth both in vitro [51] and in vivo [7, 32]. This explains the widespread use of SCs as seed cells of choice for artificial nerve grafts. However, the direct use of SCs is fraught with impediments, notably, their scanty yields. As a consequence, the motivation for the continued efforts to find alternative cells for transplantation in peripheral nerve repairs remains alive.

Earlier studies showed that the tissue-engineered artificial nerves containing bone marrow-derived stem cells (BMSCs) are useful for treating peripheral nerve defects safely [32, 62, 63]. In addition, our earlier studies demonstrated that the chemically extracted acellular nerve allograft (CEANA) containing BMSCs confers better sciatic nerve regeneration than the acellular graft alone [62, 63]. These BMSCs have been featured as an alternative source of stem cells for peripheral nerve tissue regeneration after injury because of their capacity to differentiate into SCs and enhance axonal regeneration [28, 62–64]. However, obtaining BMSCs from donors is a painful process that, in most instances, requires general or spinal anesthesia and yet presents with a low cell count yield [67]. Therefore, there is a pressing need to search for alternative stem cell sources.

Adipose tissue has been identified as an alternative source of stromal cells capable of differentiating into mesodermal cell lineages [15, 66]. Adipose-derived stem cells (ADSCs), like BMSCs, are multipotent. They are thought to have phenotypic and gene expression profiles similar to those of BMSCs. They also possess neurotrophic properties and are able to differentiate into multiple cell lineages [36, 38, 59]. The ADSC yield and proliferative ability are greater than those of BMSCs [24, 37, 61]. Therefore, this study was designed to find out if ADSCs are a good candidate for use in cell transplantation therapies of rat sciatic nerve defects ahead of BMSCs and autografts.

## Methods

### Animals

The 8-week-old adult female Sprague–Dawley rats (200–250 g) used in this study were purchased from the Experimental Animal Center of the Guangzhou University of Chinese Medicine. The rats had access to a standard rat diet and water ad libitum. They were housed under a 12-h light/dark cycle throughout the study time. All surgical procedures were conducted in accordance with the animal ethics guidelines of the National Health and Research Institutes. The experiments were approved by the Guangzhou University of Chinese Medicine’s Animal Experimentation Ethics Committee.

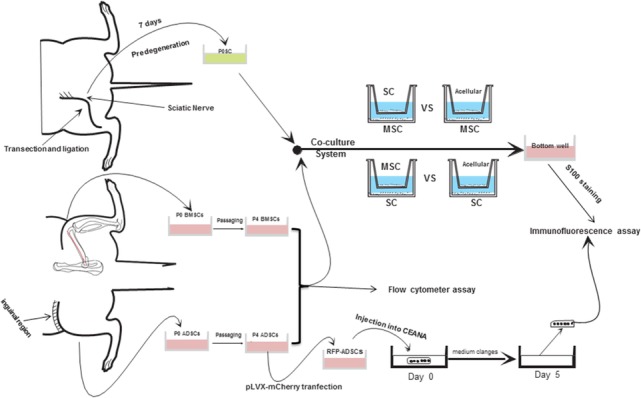



### Cultivation of Schwann cells

SCs were harvested from adult rat pre-degenerated sciatic nerves according to established procedures [62–64]. The sciatic nerve on the right side was exposed proximally opposite the sciatic notch and cut. The proximal stump was ligated with a 3-0 silk ligature to prevent regenerating axons from re-innervating the distal nerve stump during the pre-degeneration period. After 7 days of pre-degeneration, the nerve on the right side was re-exposed and an 18–20-mm-long nerve piece was removed from the distal nerve stump. Upon removal, the epineural sheaths around the sciatic nerves were removed and then the nerves were cut into 1-mm-long sections. The nerve pieces were then serially washed in Hank’s Balanced Salt Solution three times. The nerve pieces were then placed in a mixture of 0.05% collagenase and dispase enzymes (Roche, Switzerland, Cat#11097113001) in DMEM/F12 (Gibco, USA, Cat# 10565018) for 5 h followed by a 10 min spin at 1200 rpm. The pellets were re-suspended in DMEM/F12 containing 10% FBS (Gibco, USA, Cat#12662029), 1% penicillin/streptomycin (BioBasic, China), 2 mM forskolin (0.8 ng/l; Sigma, USA, Cat#F6886), and 20 ng/l bovine pituitary extract (Sigma, USA, Cat#P1476). Finally, the dissociated SCs were seeded into 6-well plates pre-coated with laminin (2 μg/ml) and poly-d-lysine (30 μg/ml Sigma, USA) and incubated at 37 °C and 5% CO_2_, with culture media changes performed after every 2 days. After the cultures reached confluence, they were passaged, split, and re-plated for co-culture procedures.

### Adult rat mesenchymal stem cell (MSC) cultures

#### Bone marrow-derived stem cell extraction

BMSCs were obtained following previous methods [62–64], with some modifications. Briefly, fresh complete bone marrow from the femoral bones of adult female SD rats (*n* = 12) was flushed using a 10-ml syringe preloaded with mesenchymal stem cell medium (MSCM, Sciencell, USA, Cat#7501) containing 100 U/ml penicillin/streptomycin solution, 5% fetal bovine serum (FBS, Sciencell, USA, Cat#0025), and mesenchymal stem cell growth supplement (MSCGS, Sciencell, USA, Cat#7552). After the cells were incubated at 37 °C in a humid atmosphere with 5% CO_2_ for 48 h, the medium was replaced and the non-adherent cells were removed. When the cells reached 80% confluence, they were passaged, split, and replated. The fourth passage (P4) BMSCs were subsequently used for induced differentiation and graft procedures.

#### Adipose-derived stem cell extraction

The inguinal areas of adult female SD rats (*n* = 10) were dissected to obtain fat tissue (~ 10 g). The harvested fat tissue was immediately rinsed with saline. After removal of the small vessels, the fat tissues were mechanically dissevered and then digested with 0.15% (w/v) collagenase I (Sigma, USA) at 37 °C for 40 min. Following digestion, the suspension was filtered through a 70-μm nylon mesh to remove undissociated tissue debris. The cell suspension was centrifuged at 1300 rpm for 5 min, and then its supernatant was discarded. The stromal cell pellet was suspended in MSCM (Sciencell, USA, Cat#7501) containing 100 U/ml penicillin/streptomycin solution, 2 mM GlutaMAX™ (100×) (Gibco, USA, Cat#35050061), 10 ng/ml epidermal growth factor (Sigma, USA), 5% FBS (Sciencell, USA, Cat#0025), and MSCGS (Sciencell, USA, Cat#7552) and was incubated at 37 °C in a humid atmosphere with 5% CO_2_. When the cells got to 80% confluence, they were washed with 0.01 M PBS buffer and treated with 1 ml 0.25% trypsin/ethylenediaminetetraacetate acid (EDTA) for 3 min. Each time the cells reached confluence, and they were passaged, split, and replated until the fifteenth cycle. P4 ADSCs were subsequently used for induced differentiation, transfection, and transplantation procedures.

#### Flow cytometry analysis of adult rat mesenchymal stem cells

Adult rat ADSCs and BMSCs at the fourth passage times were harvested using 0.25% trypsin and 0.02% EDTA. After being washed with 0.01 M PBS, the 50-ml suspensions at a cell concentration of 10^6^/ml were used as the test set. The test sets were incubated with mouse anti-rat CD90-PE OX-7 antibody (BD Pharmingen™, USA, Cat#551401), mouse anti-rat CD44H-FITC OX-49 antibody (BD Pharmingen™, USA, Cat#550974), mouse anti-rat CD45-PE-CY™5OX-1 antibody (BD Pharmingen™, USA, Cat#559135), or hamster anti-rat CD29-FITCHa2/5 antibody (BD Pharmingen™, USA, Cat#555005). After incubation in darkness for 20 min at room temperature, the cells were analyzed immediately using a flow cytometer (BD FACSCalibur; Becton Dickinson, Franklin Lakes, NJ, USA). The experiments were repeated three times. Lysis II software (Becton Dickinson) was used for data capture and analysis.

### Co-culture of MSCs and SCs

The co-culture system, which consisted of a lower six-well plate and an upper Transwell cell insert (0.4 μm pore size, BD Falcon, USA, Cat#353090), was built to juxtapose the MSCs and SCs as previously described [62, 64]. In this study, two co-culture models were established as follows: the first model was used to assess the proliferation of SCs—the SCs were seeded into the lower six-well plates (2 × 10^5^ cells/9.6 cm^2^) while the MSCs were plated on the upper Transwell cell inserts (1 × 10^5^ cells/9.6 cm^2^); the second model was used to assess the differentiation of MSCs by seeding MSCs into the lower six-well plate at a density of 1 × 10^5^ cells/9.6 cm^2^, with SCs grown on the upper Transwell cell inserts at a density of 2 × 10^5^ cells/9.6 cm^2^. The co-culture medium was MSCM medium (Sciencell, USA, Cat#7501) containing 100 U/ml penicillin/streptomycin solution, 5% FBS (Sciencell, USA, Cat#0025), and MSCGS (Sciencell, USA, Cat#7552). Control cultures of either MSCs or SCs were plated in a similar apparatus devoid of any cells in the upper Transwell cell inserts. The duration of the co-culture and control cultures was 4 days.

### Immunofluorescence staining

The SC marker, S-100, was used to identify presumptive Schwann cells after MSCs and SCs co-culture differentiation and proliferation. Immunostaining was performed according to the previous protocols [62, 64]. Briefly, cells cultured on the lower six-well plate were fixed with 4% paraformaldehyde in 0.01 M PBS for 10 min. The fixed cells were permeabilized using 0.05% Triton X-100 for 10 min and then blocked using 5% goat serum for 1 h in order to mask nonspecific binding sites. Cells were then incubated with an S100 antibody (1:2000, Dako, UK, Cat#Z0311) at 4 °C overnight. On the following day, the cells were washed three times with PBS and then incubated with corresponding secondary antibodies (1:500, conjugated to AlexaFluor 488, Invitrogen, USA) for 2 h at room temperature. After second rinsing in PBS, the cells were incubated in 4-6-diamidino-2-phenylindole-dihydrochloride (DAPI, 1:2000) to counterstain cell nuclei, for 5 min at room temperature. Finally, specimens were washed three times in 0.01 M PBS, for 5 min each time, and were then mounted with an antifade solution. Labeled cells were examined under a fluorescence microscope (Axiphot; Zeiss, Oberkochen, Baden-Wurttemberg, Germany). Image-Pro Plus (Media Cybernetics, USA) was used to acquire and process the images.

### Recombinant lentivirus packaging of pLVX-mCherry

The plasmid, pLVX-mCherry, was ordered from Biowit Technologies, Shenzhen, China. pLVX-mCherry was introduced into recombinant lentivirus by plasmid transfection. The amplification, purification, and titer evaluation of the virus were performed according to the manufacturer’s instructions. The recombinant lentivirus, pLVX-mCherry, was obtained in a total volume of 7 ml, and the viral titer (transducing units/ml) was 2.78 × 10^10^ IU/ml as determined by counting RFP-positive HEK293T cells transfected with serial dilutions of the concentrated viral culture supernatant. The lentiviruses were divided into 20 μl aliquots and used immediately or stored at − 80 °C.

### pLVX-mCherry infection of adult SD rat ADSCs

The fourth-generation adult SD rat ADSCs were evenly plated into the culture wells. When the cells in the culture wells reached 70–80% confluence, the old culture medium was removed and the fresh complete culture medium containing pLVX-mCherry lentivirus was added to the culture well until it evenly covered its bottom. The volume of pLVX-mCherry that was added into the well was calculated according to the cell number and multiplicity of infection. The cells were incubated at 37 °C overnight. On the following day, the fresh complete culture medium was added, and the cells cultured at 37 °C and 5% CO_2_ for another 24 h. The fluorescence signal of RFP was observed at 568-nm wavelength under a fluorescence microscope (MBF; Nikon, Osaka, Japan) to detect the positive rate of pLVX-mCherry-infected cells. The normal ADSCs without the pLVX-mCherry transfection served as a negative control.

### Preparation of chemically extracted acellular nerve allograft and in vitro construction of tissue-engineered artificial nerve grafts

The CEANA was prepared according to previous protocols [29, 62, 63] with the following modifications. Briefly, 30-mm sciatic nerve segments from both hind limbs were harvested under sterile conditions. Fat and connective tissues were dissected off the nerve segments and agitated in deionized distilled water (ddH_2_O) for 7 h. The nerve segments were immersed in 4% TritonX-100 (Sigma, USA, Cat# T8787) in ddH_2_O overnight. After washing in PBS (for 10 min 3 times), the nerve segments were agitated in 4% sodium deoxycholate (Sigma, US, Cat# D6750) in ddH_2_O for 24 h. The above steps were repeated twice, separated by washing steps. Every sciatic nerve segment was cut into 1.0-cm-long pieces before performing the last 3 washes in 0.01 M PBS containing 100 U/ml penicillin/streptomycin solution for 6 h. A single piece of allograft was randomly selected from the above CEANA pool and cut into 10-μm-thick longitudinal or transverse sections on a freezing microtome (HM400E; Microm, Walldorf, Germany). The sections were incubated with an S100 antibody (1:500, Dako, UK, Cat#Z0311) and mouse anti-neurofilament 200 (NF200) (1:400, Sigma-Aldrich, USA, Cat# N0142) at 4 °C overnight. This was followed by a reaction with corresponding secondary antibodies (1:500, conjugated to AlexaFluor 568 and AlexaFluor 488, Invitrogen, USA) for 2 h at room temperature. The slices were subsequently observed under a fluorescence microscope (MBF; Nikon, Osaka, Japan). The normal nerve sections served as a control. The other CEANA segments were subsequently stored in 0.01 M PBS at − 80 °C until use. Immediately before nerve graft transplantations, BMSCs, ADSCs, and pLVX-mCherry-infected RFP-ADSCs were harvested from each in a culture plate using 0.25% trypsin and then suspended in fresh complete MSCM at a concentration of 1 × 10^6^ cells/100 μl. Seven microliters of cell suspension was injected into the CEANA using a glass micropipette under a surgical microscope at × 10 magnification in order to construct three types of tissue-engineered artificial nerve grafts namely ADSCs graft, BMSCs graft, and the RFP-ADSCs graft. Fresh complete MSC culture medium was set as the control according to previously established procedures [62, 63]. The nerve grafts, with or without cells, were then incubated in fresh complete MSCM in a humidified atmosphere with 5% CO_2_ at 37 °C for 5 days. On the fifth day, the first two grafts above were quickly used for in vivo experiments while the RFP-ADSCs graft was examined by fluorescence microscopy.

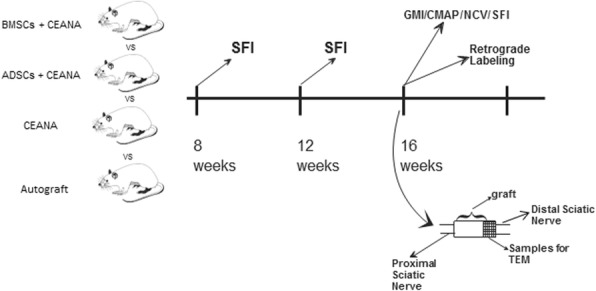



### Transplantation of tissue-engineered artificial nerve grafts

Animal surgery was conducted as previously described [62, 63]. The SD rats were anesthetized with a mixture of ketamine (80 mg/kg) and xylazine (8 mg/kg) by intraperitoneal injection. The skin on the right lateral thigh was cut open and underlying muscles pulled apart in order to expose the sciatic nerve. An 8-mm segment of the mid-thigh sciatic nerve was removed (near the obturator tendon), leaving a 10-mm-long gap after the nerve retraction. A total of 64 rats were randomly divided into four groups (*n* = 16 each group), referred to as BMSCs (sciatic nerve defect + CEANA containing BMSCs), ADSCs (sciatic nerve defect + CEANA containing ADSCs), CEANA (sciatic nerve defect + CEANA), and autograft (sciatic nerve defect + reverse autologous nerve) group. In the cell treatment groups, the 10-mm-long CEANA loaded with either P4 BMSCs or P4 ADSCs was connected to the sciatic nerve and filled the gap. In the CEANA group, the 10-mm-long CEANA loaded with an equal volume of the complete MSCM was connected to the sciatic nerve gap. In the autograft group, a 1-cm transected sciatic nerve segment in rats was reversely connected to the stumps. The wounds were subsequently closed in layers.

### Functional assessment of re-innervation

#### Sciatic Functional Index (SFI)

Walking track analysis was performed at 8, 12, and 16 weeks after surgery, based on the Bain-Mackinnon-Hunter (BMH) Sciatic Functional index-formula [3], a method previously used by our group [62, 63]. The SFI was calculated according to the equation:

$$ \mathrm{SFI}=-38.3\times \left(\mathrm{EPL}\hbox{-} \mathrm{NPL}\right)/\mathrm{NPL}+109.5\times \left(\mathrm{ETS}\hbox{-} \mathrm{NTS}\right)/\mathrm{NTS}+13.3\times \left(\mathrm{EITS}\hbox{-} \mathrm{NITS}\right)/\mathrm{NITS}\hbox{-} 8.8 $$

where toe spread (TS) is the length between the first and fifth toes; print length (PL) is the length between the third toe and heel, and the second and fourth toe spread (intermediary TS [ITS]) was measured on the experimental side (E) and the contralateral normal side (N) in each rat. In general, the SFI ranges from 0 for normal nerve function down to − 100 for total dysfunction. The SFI is a negative value and the higher the SFI the better the function of the sciatic nerve. SFI was obtained for each group (BMSCs, ADSCs, CEANA, and autograft group).

#### Electrophysiological measurement

At 16 weeks after surgery, the rats (*n* = 8) in each group were subjected to electrophysiological tests immediately after completing gait evaluation using a method described previously [62, 63]. Briefly, while the rat is under general anesthesia, the previous surgical site was carefully reopened to re-expose the sciatic nerve. A stimulating electrode was placed under the sciatic nerve proximal to the graft. The compound muscle action potential (CMAP) was recorded with a recording needle placed in the gastrocnemius muscle 10 mm below the tibial tubercle. The CMAP recorded on the contralateral uninjured side was used as the normal control. Electromyography (EMG) was performed by inserting a recording electrode into the belly of the gastrocnemius muscle. The latency and amplitude of the EMG were obtained. The distance between the proximal and distal stimulation sites was also measured in order to enable calculation of the regenerated sciatic nerves’ nerve conduction velocity (NCV).

#### Gastrocnemius Muscle Index (GMI)

Immediately following the electrophysiological measurements, gastrocnemius muscles were carefully harvested from contralateral and injured sides and immediately weighed while still wet in order to determine the wet mass ratio of muscles (the wet mass of muscle from the injured side/the wet mass of muscle from the contralateral side).

#### Retrograde labeling and labeled cell number counting

Guided by the 10-0 suture at the proximal and distal ends, the sciatic nerves in each of the remaining rats (*n* = 8 in each group) were re-exposed 16 weeks after surgery. Around 0.8 μl of 2% biotinylated dextran amine (BDA) was slowly injected into the graft at a site 5 mm from the distal end, using a 10-ml Hamilton syringe with a sharpened needle tip, as described previously [62, 63]. Four days later, labeled rats were sacrificed and perfused with 4% paraformaldehyde in 0.1 M phosphorylated buffer. The L4-5 segments of the spinal cords were collected and sectioned longitudinally, at a thickness of 25 μm. Section observation was carried out under the fluorescence microscope (BX51; Olympus, Tokyo, Japan). Labeled cells in the L5 segment were counted on every second section.

#### Transmission electron microscopy

The regenerated nerves of BMSCs, ADSCs, CEANA, and autograft groups were harvested at 16 weeks after surgery and fixed overnight in 2.5% glutaraldehyde in 0.1 M PB, followed with overnight fixation using 1% osmium tetroxide. The tissue was then dehydrated in graded ethanol (30%, 50%, 70%, 80%, 90%, and 95% for 5 min each; and 100% for 3 times, 30 min each time). The tissue was then infiltrated with propylene oxide (PO) twice, for 30 min each time. PO to Epon (1:1) for 1 h was followed by pure Epon overnight. After that, the nerve tissues were embedded in Epon, cut into 0.5-μm semi-thin sections or 60-nm-thick ultrathin sections, and then stained with 0.5% toluidine blue in 1% borax or 3% uranyl acetate and 1% lead citrate, respectively. The semi-thin sections were observed under a light microscope while the ultrathin slices were observed under a transmission electron microscope (JEOL Ltd., Tokyo, Japan). The images were taken from 10 random fields of each section. Image-Pro Plus software (Media Cybernetics, Rockville, MD, USA) was used to determine the diameter of myelinated nerve fibers and the thickness of myelin sheaths.

### Statistical analysis

Experimental results were expressed as mean ± standard deviation. Statistical comparisons were performed using GraphPad Prism 6.0 software. The comparisons of two groups or time points were done using a non-paired student *t* test. Statistical significance was determined by ANOVA in events where more than 2 groups were compared. Statistical significance was set at *p* < 0.05.

## Results

### Adult primary SC characteristics

**Fig. 1:**
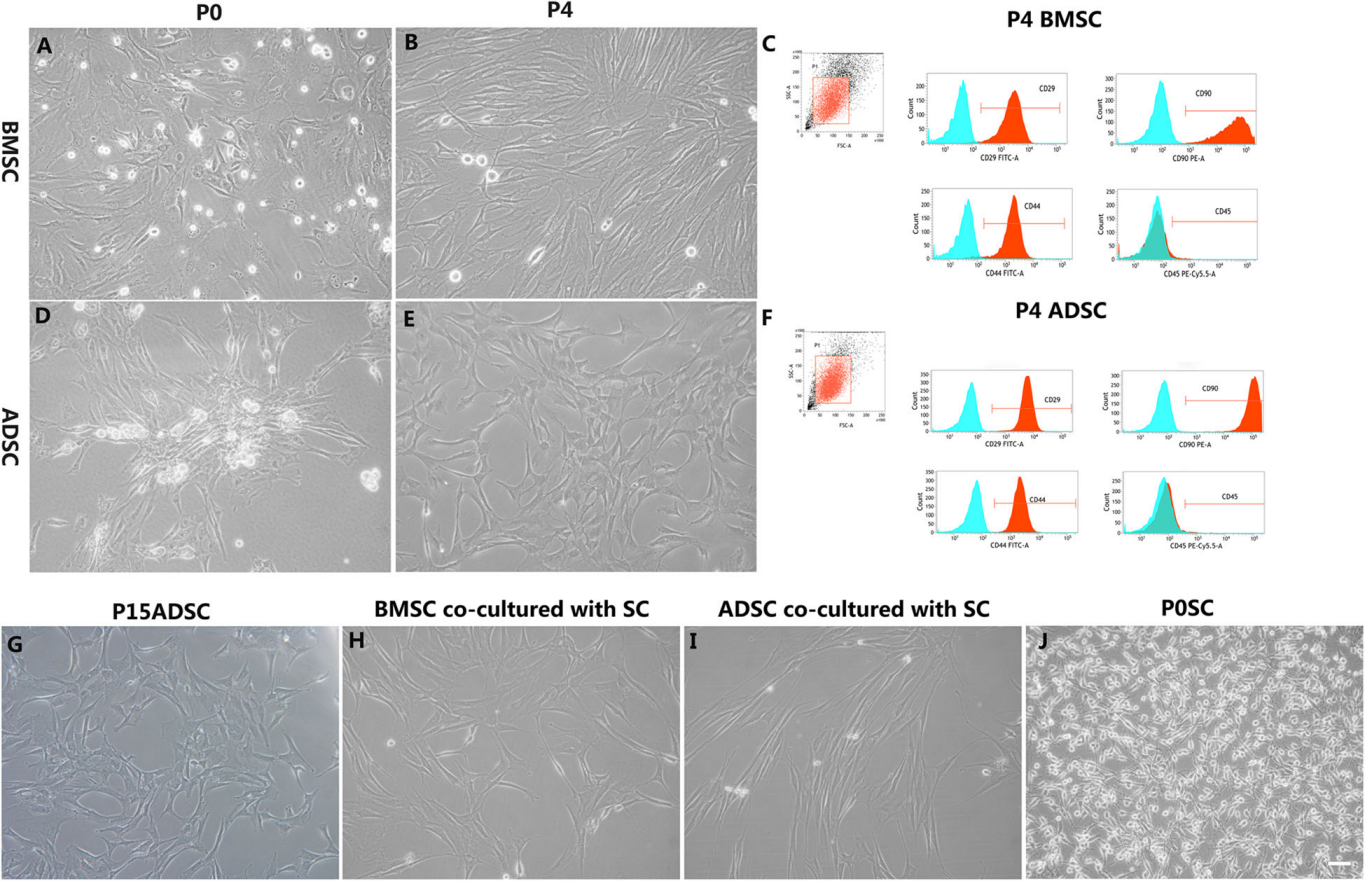
The morphology and identity of various cells under study as analyzed using phase-contrast microscopy and flow cytometry. Phase-contrast micrographs showing the morphology and flow cytometric analysis of adult mesenchymal stem cell (MSCs) (**a**–**f**). **a** Harvested P0 BMSCs. **b** Fourth passage (P4) BMSCs. **c** Flow cytometric analysis of P4 BMSCs. **d** P0 ADSCs. **e** P4 ADSCs. **f** Flow cytometric analysis of P4 ADSCs; scale bar, 20 μm. MSCs change morphology when co-cultured with SCs (**g**–**j**). **g** P15 ADSCs. **h** P4 BMSCs co-cultured with Schwann cells (SCs) for 4 days. **i** P4 ADSCs cultured with SCs for 4 days. **j** Phase-contrast micrograph showing the morphology of adult primary SCs (P0); scale bar, 20 μm

Our results showed that the primary adult SCs typically exhibited bipolar and occasionally multipolar spindle-shape (Fig. 1j and Fig. 3a). The proportion of Schwann cell marker (S100) positive cells was 71 ± 1.9% at the time of confluence (Fig. 3b, c).

### Characteristics of adult MSCs in vitro prior to co-culture

Adult primary BMSCs obtained from the bilateral femurs of adult male rats were heterogeneous in morphology exhibiting a combination of small rounded, spindle-shaped, or large flattened cells (Fig. 1a). During subsequent passages, we observed the disappearance of the small rounded shape as the cells gradually assumed a more fibroblast-like appearance. From P4, the fibroblast-like morphology became predominant (Fig. 1b), an observation consistent with previous studies on BMSCs [62–64]. Flow cytometric analysis showed that the passage 4 BMSCs were positive for the well-defined rat mesenchymal stem cell (rMSC) markers CD29, CD90, and CD44H with greater than 97% purity (Fig. 1c). Adult primary ADSCs obtained from the inguinal region adipose tissue of adult female rats showed colony-like distribution coupled with swirling growth (Fig. 1d). The adult rat ADSCs within 3–5 passages appeared as an adherent monolayer of large and flat cells without cytoplasmic extensions (Fig. 1e). They were easily expanded up to 15 passages while maintaining the undifferentiated state with a spindle-shaped, fibroblastic morphology (Fig. 1g). However, just like their BMSC counterparts, the 4th passage ADSCs tested positive to the well-defined markers of rat mesenchymal stem cell (rMSC) markers CD29, CD90, and CD44H with greater than 99% purity, but negative for the hemopoietic surface antigen CD45 (Fig. 1f).

### Induction of MSC differentiation in SC co-culture system

**Fig. 2:**
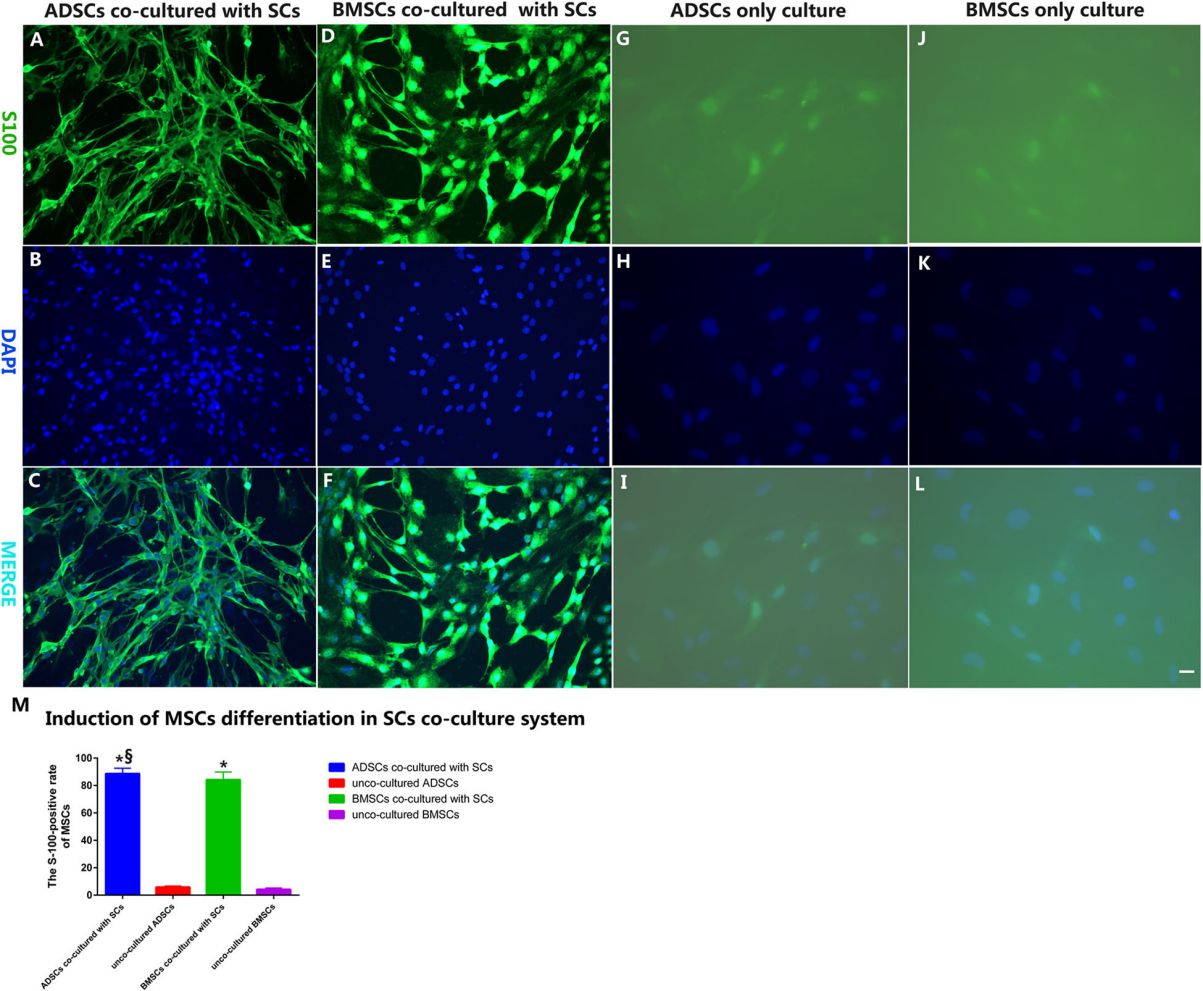
MSCs co-cultured with SCs. Scale bar, 20 μm. Morphological analysis and distribution of MSCs co-cultured with SCs or acellular control evaluated after 4 days as shown by immunofluorescence imaging with anti-S00 (green, **a**, **d**, **g**, **j**) and DAPI (blue, **b**, **e**, **h**, **k**) and their merged micrographs (**c**, **f**, **i**, **l**). **a**–**c** ADSCs co-cultured with SCs. **d**–**f** BMSCs co-cultured with SCs. **g**–**i** ADSC-only culture (control). **j**–**l** BMSC-only culture (control). Histogram (**m**) showing comparisons of the number of S100-positive cells as a percentage of DAPI-positive nuclei in the SCs-MSC co-culture system. *p < 0.05 versus single-cell/mono-cultured group, ^§^*p* > 0.05 versus BMSC group

A greater number of the co-cultured BMSCs exhibited the typical glial differentiation morphology: a fusiform shape with a centrally placed rounded nucleus along with two or three slender processes (Fig. 1h and Fig. 2d–f). The majority of co-cultured ADSCs became increasingly elongated spindle-shaped with two- or three-polar processes. They also displayed obvious retraction of the cytoplasm towards the nuclei, forming compact cell bodies with cytoplasmic extensions and proportionally large nuclei similar to that of Schwann cells. The characteristic morphology is shown in Figs. 1i and 2a–c. These fusiform-shaped, spindle-like cells were seen to continue proliferating, leading to a higher cell density. On the other hand, the mono-cultured BMSCs or ADSCs just maintained their mesenchymal morphology (Fig. 2g, j). The densities of BMSCs or ADSCs co-cultured with SCs were much higher than observed in control mono cultures, as shown by S-100 (Fig. 2a, d, g, j) and DAPI (Fig. 2b, e, h, k) double labeling (Fig. 2c, f, i, l). Quantitative evaluation of the MSC differentiation into SCs using immunocytochemical staining revealed that the BMSC–SCs positive for S100 (84.23 ± 5.65%) and ADSC–SCs positive for S100 (88.6 ± 4.0%) were much higher than the 4.14 ± 1.03% and 5.76 ± 0.89% of MSCs–SCs positive for S100 in the acellular control cultures. These differences were statistically significant at *p* < 0.05. However, the difference between BMSC and ADSC co-culture groups was not statistically significant (*p* > 0.05) (Fig. 2m).

### State of SC proliferation under the MSC co-culture system

**Fig. 3:**
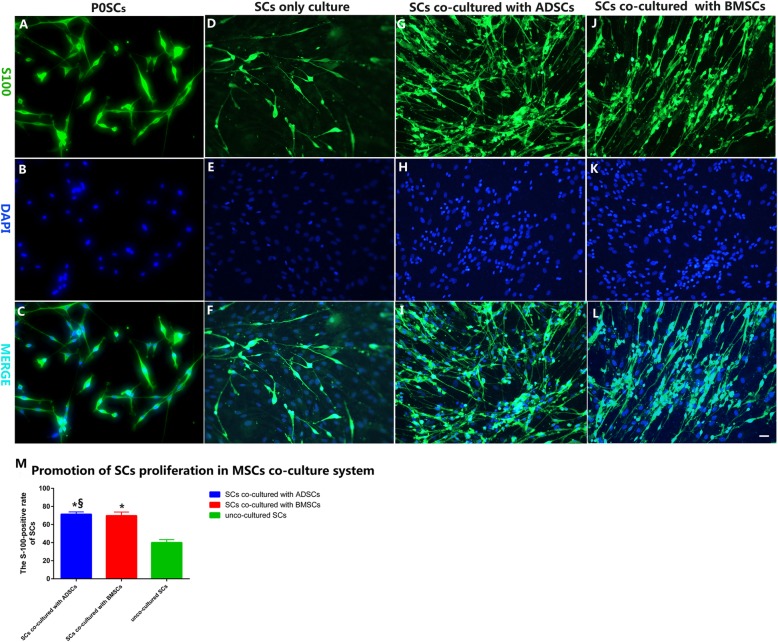
SCs co-cultured with MSCs. Scale bar, 20 μm. Morphological analysis and distribution of adult primary SCs. SCs were co-cultured with either MSCs or as an SC-only control for 4 days. In the SC–MSC co-culture system as shown by immunofluorescence imaging with anti-S00 (green, **a**, **d**, **g**, **j**) and DAPI (blue, **b**, **e**, **h**, **k**) and their merged micrographs (**c**, **f**, **i**, **l**): **a**–**c** primary SCs; **d**–**f** SC-only culture (control); **g**–**i** SCs co-cultured with ADSCs; **j**–**l** SCs co-cultured with BMSCs. Histogram (**m**) comparing the number of S100-positive cells as a percentage of DAPI-positive nuclei in the SCs-MSCs co-culture system. *p < 0.05 versus SC-only culture group, ^§^*p* > 0.05 versus BMSCs group

As seen in Fig. 3i, l (S-100 and DAPI double labeling), SCs exhibited a fusiform body with two to three thin and elongated processes when co-cultured with MSCs. S-100-labeled cells (Fig. 3g, j) were more in the ADSC or BMSC co-cultured system than in the control mono cellular cultures (Fig. 3d). Quantitatively, we noted that 69.76 ± 4.013% of the DAPI-labeled nuclei were S-100-positive in BMSC co-cultures (Fig. 3j, l). This result is consistent with those reported previously in similar co-cultures [62, 64]. The percentage was 71.35 ± 2.59% in the ADSC co-cultures (Fig. 3g, i). These findings were significantly higher than the 40.03 ± 3.23% counted in acellular control cultures (Fig. 3d, f) (*p* < 0.05). However, the difference between the BMSC and ADSC co-culture groups was not statistically significant (*p* > 0.05) (Fig. 3m).

### The characterization of the CEANA and in vitro construction of tissue-engineered artificial nerve grafts

**Fig. 4:**
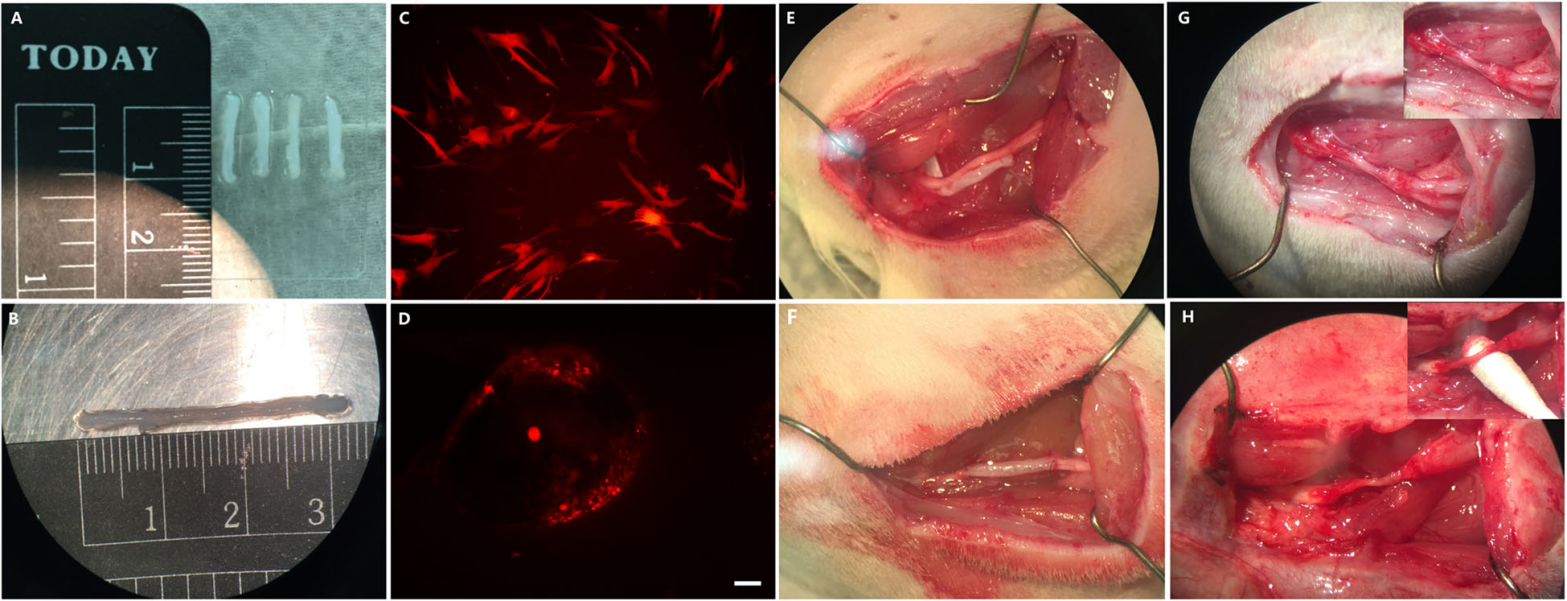
CEANA gross morphology, cell transplantation, and its state at 16 weeks after surgery. **a** Milky, semi-transparent chemical-extracted acellular nerve allograft (CEANA). **b** The scaffold containing transplanted P4 RFP-ADSCs. **c** Immunofluorescence of pLVX-mCherry-transfected P4 ADSCs (red); scale bar, 20 μm. **d** Immunofluorescence of the CEANA infused with P4 RFP-ADSCs and cultured for 5 days (red); scale bar, 20 μm. **e** The contrast of normal sciatic nerve and CEANA. **f** The CEANA containing P4 RFP-ADSCs is shown in situ immediately after transplantation. **g** CEANA infused with P4ADSCs at 16 weeks after surgery. **h** CEANA alone (no cells transplanted into it) at 16 weeks after surgery

**Fig. 5:**
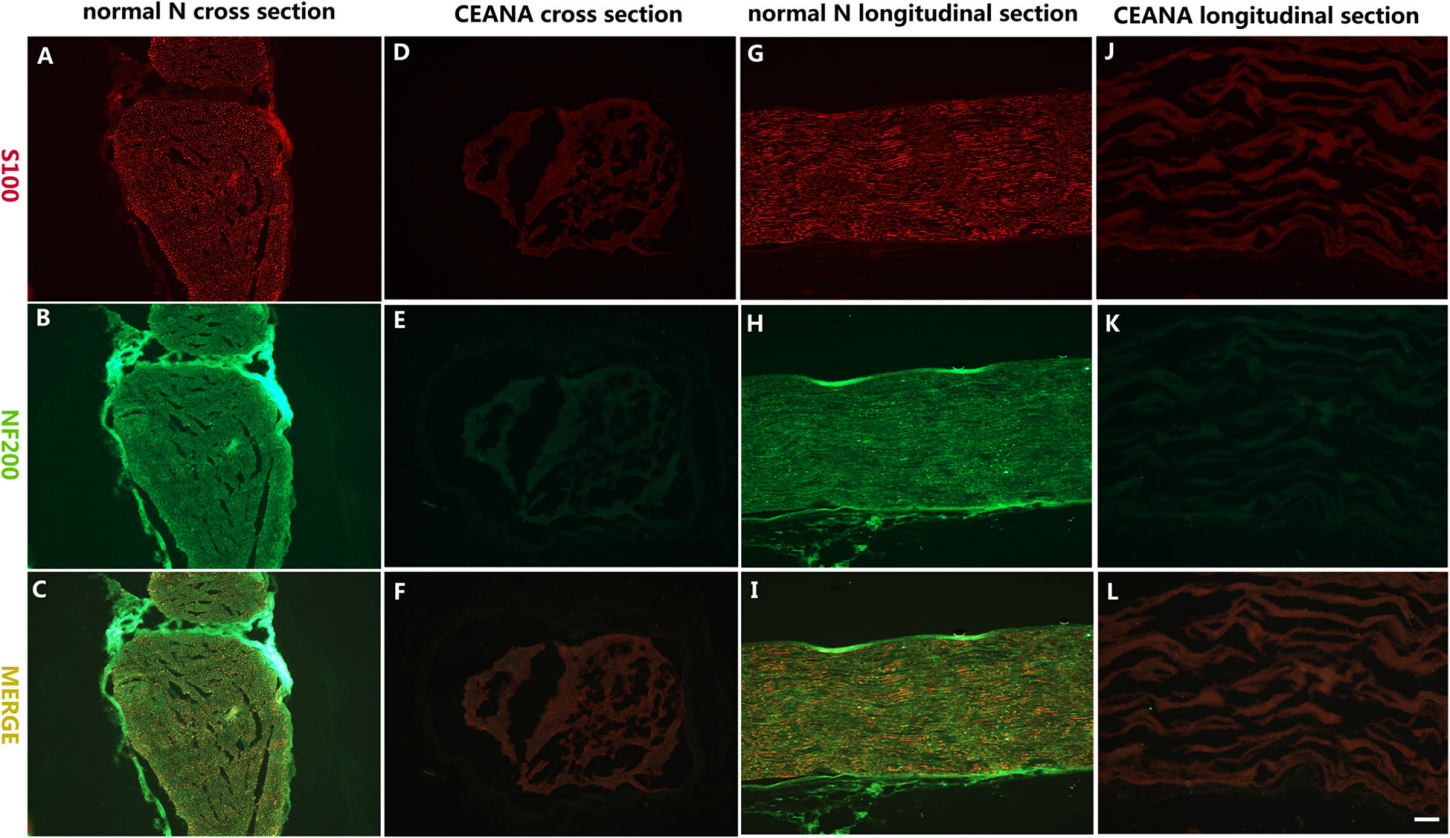
Differences between normal and chemical-extracted acellular nerve allograft. Scale bar, 20 μm. Immunofluorescence with anti-S100 (red, **a**, **d**, **g**, **j**) and with anti neurofilament 200 (anti-NF 200) (red, **b**, **e**, **h**, **k**) and their merge (**c**, **f**, **i**, **l**) for contrasting the normal nerve from chemical-extracted acellular nerve allograft (CEANA). **a–c** Cross section of normal nerve. **d**–**f** Cross-section of CEANA. **g**–**i** Longitudinal section of normal nerve. **j**–**l** Longitudinal section of CEANA

The scaffolds that were chemically extracted appeared milky, semitransparent and felt softer compared to the normal nerve (Fig. 4a, b, and e). The cross and longitudinal section fluorescent immunostaining results showed that myelin and axons were absent in the chemically extracted nerve scaffold. The tissue was also found to be loose, and the nerve axon and the myelin sheath have been removed compared with the normal nerve (Fig. 5a–l). The transplanted RFP-ADSCs (Fig. 4c) can survive for 5 days in the CEANAs when incubated in complete MSCM in a humidified atmosphere with 5% CO_2_ at 37 °C. This observation was further verified using fluorescence microscopy as shown in Fig. 4d. Generally, the ADSC-infused CEANA group grafts (Fig. 4g) were thicker than the CEANA-only grafts (Fig. 4h) at 16 weeks after transplantation. However, it was apparent that both transplants shrunk over the course of 16 weeks as shown by their relative thinness compared to the transplant time ADSC-loaded CEANA (Fig. 4f).

### Recovery of sciatic nerve function and target re-innervation

#### SFI outcome

**Fig. 6:**
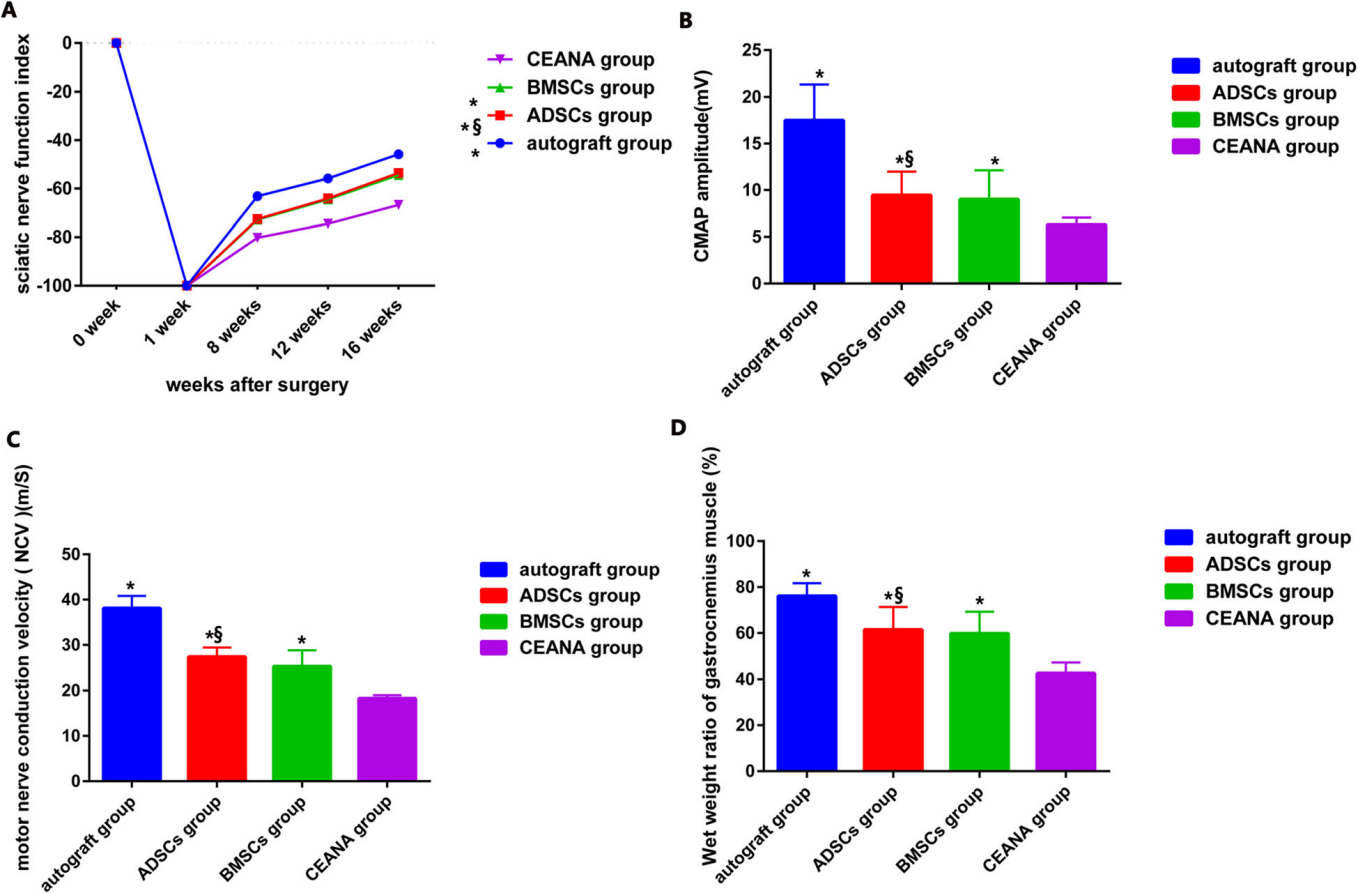
MSCs promote repair of injured sciatic nerve function. **a** Sciatic Functional index of the treated side obtained from the autograft group, ADSC group, BMSC group, and the CEANA group at 8, 12, and 16 weeks after surgery. **b** The CMAP of the treated side obtained from the autograft group, ADSC group, BMSC group, and the CEANA group at 16 weeks after surgery. **c** The NCV of the treated side obtained from the autograft group, ADSC group, BMSC group, and the CEANA group at 16 weeks after surgery. **d** The ratios of gastrocnemius mass of the treated side obtained from the autograft group, ADSC group, BMSC group, and the CEANA group at 16 weeks after surgery. *p < 0.05 versus CEANA group, ^§^*p* > 0.05 versus BMSC group

To evaluate the efficacy of ADSCs on the functional improvement of rats with peripheral nerve injury, we compared this group’s SFI with that of the BMSCs-treated, the autograft, and the CEANA groups at 8, 12, and 16 weeks after surgery. Before surgery, SFI values for all groups are normally zero. Following sciatic nerve transection, the SFI value decreased to − 100 due to the complete denervation in all rats. Our results showed that the sciatic nerve function recovery was significantly faster in ADSC and BMSC groups when compared with the CEANA group (*p* < 0.05). However, we observed that both (ADSCs/BMSCs group) were slower than in the autograft group. Moreover, the ADSC group exhibited similar SFI scores to that of the BMSC group although not statistically significant (*p* > 0.05) (Fig. 6a).

#### Electrophysiology

At 16 weeks after nerve grafting, the CMAP amplitude value and nerve conduction velocity (NVC) in the 2 cell-treated groups and the autograft group were both significantly greater than that in the CEANA group (all *p* < 0.05). Further analysis revealed that both the CMAP amplitude and NVC of the ADSC group were similar to those obtained from the BMSC group with no significant differences (*p* > 0.05). However, both groups’ CMAP and NVC were less than those of the autograft group (*p* < 0.05) (Fig. 6b, c).

#### Muscle mass measurement

At 16 weeks after surgery, the mean ratios of gastrocnemius muscle mass were measured in the four different grafts groups. The muscle mass ratios of the 2 cell-treated groups (BMSC/ADSC) were not significantly different (*p* > 0.05). On the other hand, both cell-treated groups’ mean muscle masses were significantly larger than the CEANA group’s (*p* < 0.05) but lower than in the autograft group (*p* < 0.05) (Fig. 6d).

#### Biotinylated dextran amine staining

**Fig. 7:**
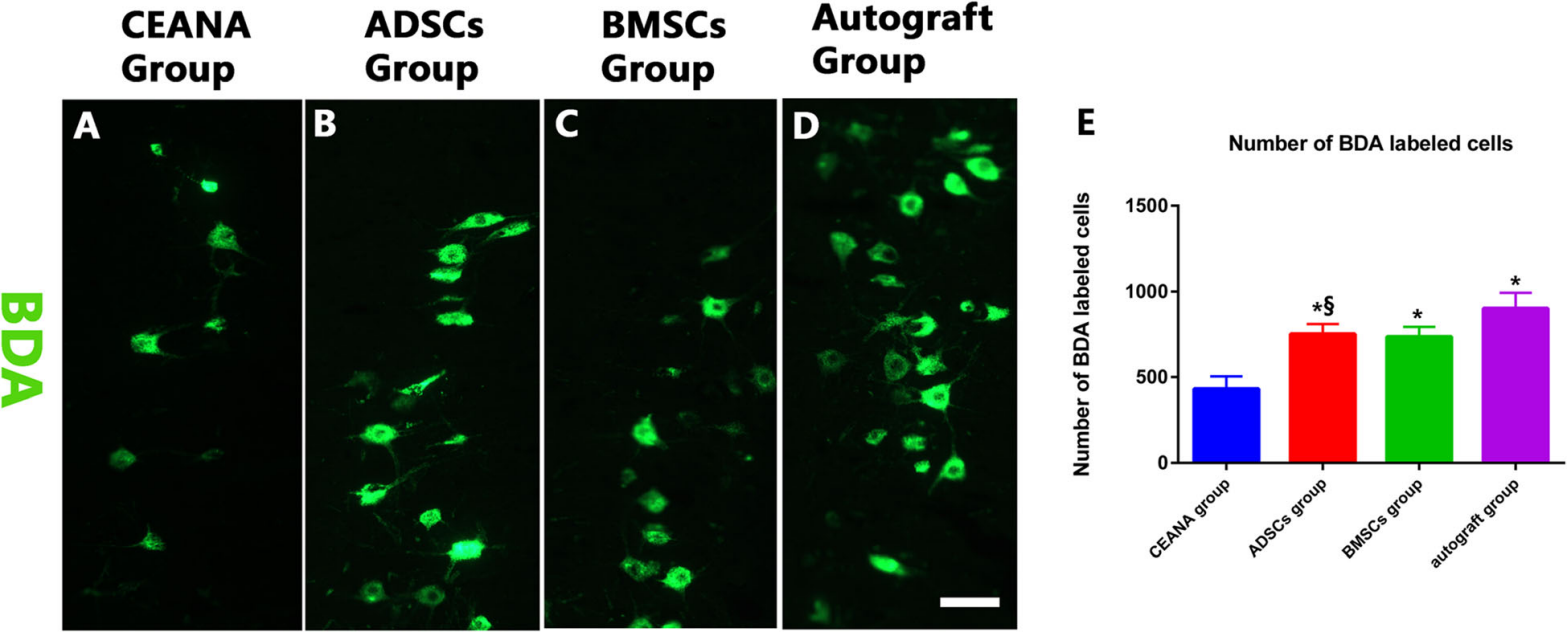
Immunofluorescence showing BDA (green) retrogradely transported to ventral horn motor neurons. Scale bars, 50 μm. **a**–**d** The longitudinal sections of L4–L5 spinal cord segments at 16 weeks after nerve grafting in the autograft group, ADSC group, BMSC group, and the CEANA group, respectively. **a** CEANA group. **b** ADSC group. **c** BMSC group. **d** Autograft group. Histogram. (**e**) A comparison of the number of BDA-labeled cells of **a**–**c** groups. *p < 0.05 versus CEANA group, ^§^*p* > 0.05 versus BMSC group

Next, we sought to find out if the observed functional improvements were associated with a rise in the number of regenerating motor neuron axons, especially into the distal peripheral nerve. We performed retrograde labeling studies by injecting BDA into the lower end of the graft and counted labeled cells in the ventral horn of the spinal cord. There was no statistically significant difference in the number of retrogradely labeled cells between the two cell-treated groups (*p* > 0.05). However, these two cell-treated groups (BMSC/ADSC) had a significantly higher number of labeled cells than the CEANA group (*p* < 0.05). On the contrary, both BMSC and ADSC cell-treated groups’ cell counts were less than those in the inverted autograft group (*p* < 0.05) (Fig. 7a–e).

#### Morphological and morphometric analysis of regenerated nerves

**Fig. 8:**
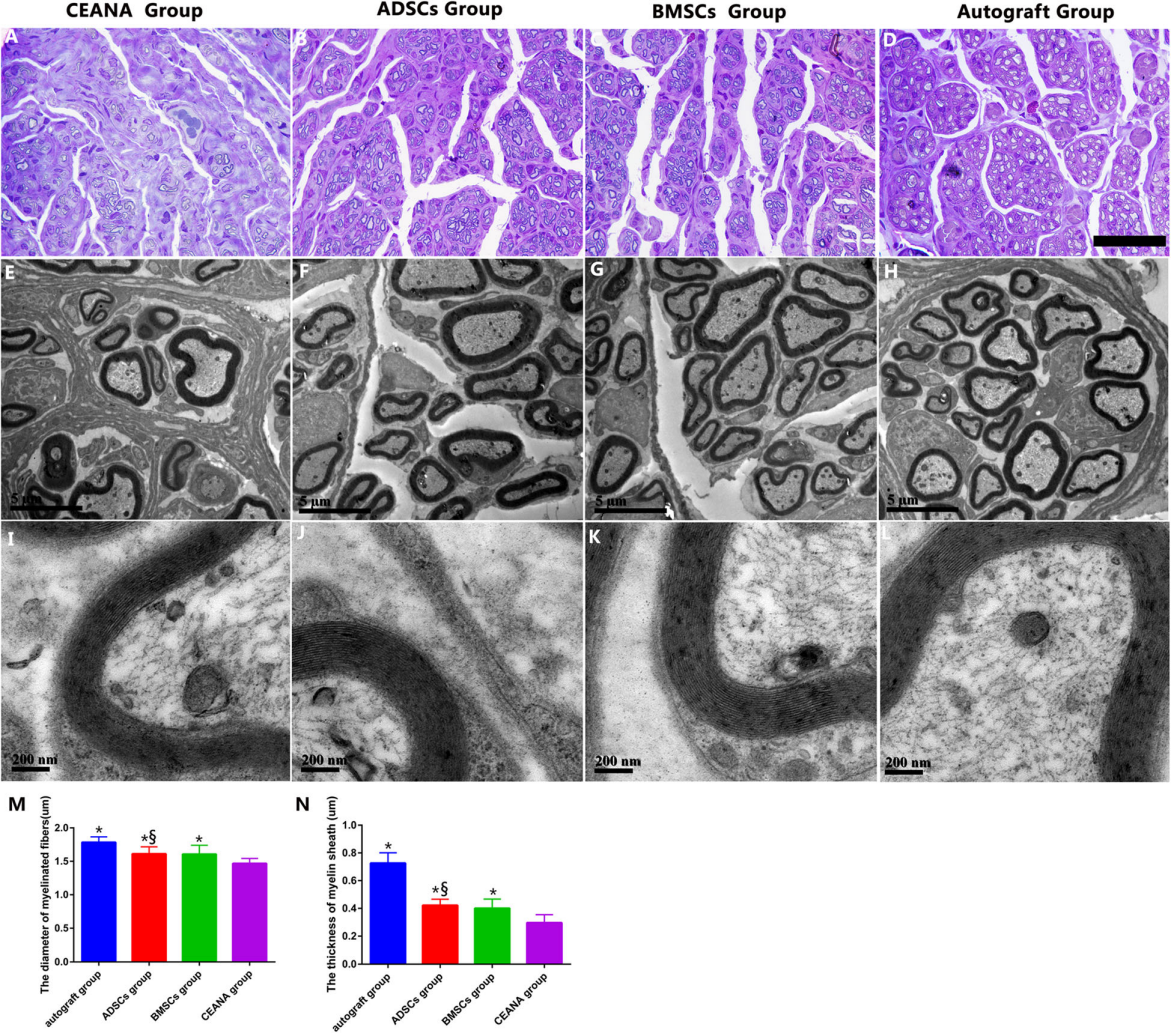
Representative images of semi thin sections (**a**–**d**) and transmission electron micrographs (**e**–**l**) of transverse sections of distal regenerated nerves taken at 16 weeks after nerve grafting from the autograft group, ADSC group, BMSC group, and the CEANA group. Scale bars, 20 μm (**a**–**d**), 5 μm (**e**–**h**), and 0.2 μm (**i**–**l**). Histograms comparing the diameter of myelinated fibers (**m**) and the thickness of myelin sheath (**n**) among the three (not 4) different groups. *p < 0.05 versus CEANA group, ^§^*p*>0.05 versus BMSC group

Figure 8b shows that the thickness of myelin and axonal diameters of the ADCS-treated group were comparable to that of the BMSC-treated group (Fig. 8c). On the other hand, the graft devoid of cellular transplantation (CEANA-only, Fig. 8a) exhibited the thinnest myelin cover and axonal diameter while the autograft group (Fig. 8d) was roundly superior. We also demonstrated quantitatively that ADSC infusion resulted in an increased diameter of axons as well as the sizes of axons elongating into the implants. The transmission electron micrographs taken 16 weeks after nerve grafting showed newly regenerating, myelinated nerve fibers coursing into the distal stump of the injured nerve. These nerve fibers had a clear, electron-dense myelin sheath and a basal SC membrane (Fig. 8e–l). Further morphometric analysis revealed that the CEANA group had the smallest axonal diameters of regenerating myelinated nerve fibers compared with the other three groups. The newly regenerated myelin sheaths were also thinner in the CEANA group (Fig. 8a, e, i) than those of the other three groups. By comparing the morphometric data noted above, we found that neither the diameter of regenerated myelinated fibers nor the thickness of the myelin sheath showed a significant difference between the BMSCs group and ADSCs groups (*p* > 0.05). However, both groups’ myelin sheaths and axons were significantly wider and thicker than observed in the CEANA group, respectively (*p* < 0.05). Overall, the autograft group had the thickest myelin sheaths and widest axons of them all (*p* > 0.05; Fig. 8m, n).

## Discussion

The present study established a reproducible method for acquiring pure and rapidly proliferating adult ADSCs from the inguinal region adipose tissue of adult female rats. We were able to easily expand the number of harvested ADSCs up to 15 passages while maintaining the distinctive undifferentiated morphology of a spindle-shaped, fibroblastic variety (Fig. 2g). Flow cytometric analysis showed that 4^th^ passage ADSCs were positive for the well-defined rat mesenchymal stem cell markers CD29, CD90, and CD44H, but negative for the hemopoietic surface antigen CD45. Therefore, these results of ADSC characterization are not only comparable with those of BMSCs in previous studies [62–64] but also surpassed them. ADSCs were generally able to maintain their morphological features and cell activity for up to the 15th passage while there was a tendency of decline in BMSCs. These results are proof that our method of extraction confirms that they are indeed rMSC and, therefore, could be explored for use as alternative stem cells in the treatment of peripheral nerve injuries.

The ideal stem cell-based source is supposed to be easily accessible, able to proliferate robustly in vitro, be multipotent, and be transplantable without eliciting host immunological reaction [4]. ADSCs have the capacity to satisfy these conditions. Developmentally, ADSCs and BMSCs are derived from the embryonic mesoderm and, therefore, share identical phenotypic as well as gene expression profiles [15, 36, 38, 59, 66]. Both cell types can differentiate into several mesenchymal tissue lineages such as adipocytes [49], osteoblasts [20], myocytes [35], chondrocytes [42], endothelial cells [2], and cardiomyocytes [65]. They can also be induced into neural lineages in vitro [9, 22, 56]. The subcutaneous adipose tissue has a higher mesenchymal cell count (2 per 100 cells) than the bone marrow (1 per 25,000–100,000 cells) [5, 16, 44, 53]. Moreover, the ADSC yield from adipose tissue and subsequent proliferation rate far exceeds that of BMSCs from the bone marrow [24, 37, 61]. In addition, ADSCs can be isolated easily by conventional liposuction procedures, which overcome tissue morbidity compared to bone marrow aspiration or Schwann cell harvesting. ADSCs also proliferate rapidly in culture. Thus, all these advantages set them as an ideal alternative cell to SCs and BMSCs for transplantation in the regeneration of injured peripheral nerves.

It was previously shown that ADSCs are inducible into SC-like cells following a protocol used to induce BMSCs into presumptive SC [36, 59]. In the current study, we found that ADSCs could be induced into Schwann-like cells which are spindle-shaped and express putative SC markers such as S-100, by indirect co-culture with SCs in vitro. The method was previously used to build a BMSC and SC co-culture system which provided two kinds of co-cultured cells with a dynamically synchronous micro-environment but prevented intercellular contact [62, 64]. We also observed that ADSCs promote the proliferation of adult SCs in the co-culture system described above. Intriguingly, we further observed reciprocal beneficial effects between ADSCs and SCs in the co-culture system in vitro. The results from this in vitro co-culture system demonstrated that SCs have a positive inductive effect on ADSCs and, in turn, ADSCs also do the same to SCs. This shows ADSCs’ significant therapeutic potential via their ability to secrete immuno-modulatory and trophic cytokines. Numerous in vivo and in vitro studies have demonstrated the paracrine activities of ADSCs through secreting high levels of cytokines of therapeutic and immuno-modulatory importance, such as IL-1Ra, IL10, and IL13 [6]; VEGF; ciliary neurotrophic factor (CNTF); and NGF [30, 34, 55]).

The proliferation and migration of SCs is a critical process inducing the regeneration of axons across a gap between nerve stumps such as caused by injury [40]. In addition, axons are known to grow only a short distance beyond their own reparative matrix while an intact endoneurium is associated with better functional outcomes. Therefore, the formation of a properly aligned extracellular matrix scaffold is essential in enhancing the proliferation and migration of seeded cells, through which blood vessels and other cell types can immigrate and form a new nerve supportive structure [40, 58]. In this present study, the CEANA was used to bridge a 1-cm sciatic nerve defect. This was done to facilitate ADSC and SC migrations as a supportive conduit which can maintain the 3-dimensional reticular structure for new axonal growth [18, 54]. More importantly, most antigenic substances in the CEANA, including nerve axon and the myelin sheath (as shown by immunofluorescence staining), have been removed by chemical extraction to minimize immune rejection. RFP-ADSCs were used to evaluate the fate of cells after being injected into CEANA. The results demonstrated that ADSCs are able to survive in CEANA for 5 days after in vitro implantation. Therefore, we showed that adult ADSCs are well compatible with the CEANA and, therefore, an excellent alternative for SCs or BMSCs in vitro (Fig. 4c, d). This marks a very important step towards future clinical translation in human patients.

Furthermore, we compared the ability of the chemically extracted acellular nerve allograft containing either BMSCs or ADSCs with autologous nerve grafts and acellular grafts to cause regeneration through a sciatic nerve defect in rats. This enabled us to determine if ADSCs are a feasible alternative stem cell source for peripheral nerve injury treatments in vivo. As shown in our results, there was a similarity in the positive functional outcomes between ADSC- and BMSC-treated groups. Therefore, we have demonstrated, in principle, that adipose tissue could be a good source of stem cells for use in nerve repair treatments. It would be interesting, in the future, to establish the underlying molecular mechanisms since these may be immunomodulatory and trophic cytokines amenable to therapeutic targeting.

Functional recovery is the ultimate aim in any therapy following peripheral nerve injury. Walking encompasses complex sensorimotor as well as cortical integration [10, 41], and walking track analysis is used to measure post-nerve repair functional recovery in rat models ([3, 19, 41]). We noted that ADSCs infused into CEANAs enabled improved motor functional recovery after transplantation surgery. Although the Sciatic Functional index reading was less than that of the autograft, the ADSC group’s level of function was comparable to routinely used BMSC treatment group. In pursuit of patient safety and comfort, further exploration of this new alternative would go a long way in enabling the timely clinical translation of ADSCs for peripheral nerve defects treatment. Despite being considered the golden standard of peripheral nerve repair, autografts cause donor site morbidity as well as the potential loss of function. As a result, the idea of an alternative stem cell source to address these, among other issues, motivated this study. Armed with this new stem cell candidate, we further determined that these functional improvements observed were associated with a rise in newly regenerating motor neurons axons into the path of the distal peripheral nerve. These biological changes in injured rats were confirmed using many replicable ways. For instance, we injected BDA into the distal part of the sciatic nerve graft and counted BDA-positive cells in the ipsilateral ventral horns of the spinal cord. Our results showed similarity in the numbers of BDA-positive cells between ADSC- and BMSC-treated groups suggesting that there was a similar rise number of regenerating axons in both groups. Additionally, cellular groups’ BDA-positive neurons were significantly more than observed in the acellular CEANA-treated group. It is therefore apparent that CEANA treatment alone did not confer any functional advantage in this rat model of sciatic nerve injury. This finding supports the continued search for alternative stem cell sources.

Furthermore, nerve conduction velocity assessments provide direct proof for the conductive function of the nerves under study [39, 41]. It is established that, generally, nerve conductive function depends on the diameter of axons and the thickness of the myelin sheath [11, 41]. In light of this, we confirmed, by light (semi-thin sections) and transition electron (ultra-thin sections) microscopy, that ADSCs infused into CEANAs facilitated the development of new and wider axons extending into the transplanted nerve grafts and proceeded distally. Target muscle re-innervation is also an important measure for peripheral nerve regeneration and is often mirrored by reduced muscle atrophy [14, 21, 43] as well as a greater degree of neuromuscular communication at the neuromuscular junction. Our study’s CMAP and gastrocnemius muscle wet mass ratio independently confirmed that ADSCs infused into CEANAs enhanced target organ re-innervation. At the end of 16 weeks after grafting, all histological and functional analysis results consistently demonstrated that ADSCs loaded into CEANA significantly enhanced the recovery to the injured sciatic nerve compared with the CEANA-only group. However, ADSCs loaded into CEANA’s beneficial effect on sciatic injury regeneration were not significantly different from treatment with BMSCs in the CEANA group. Although the exact mechanisms underpinning these beneficial effects of grafted cells are complex and most certainly multifaceted, previous work has elucidated two competing models: paracrine secretion by MSCs and differentiation of MSCs into SCs which in turn are pro-regeneration. On one end, the MSCs’ secretome through multiple mechanisms directly would support intrinsic nerve regeneration. Beyond paracrine secretion by MSCs/ADSCs, on the other hand, some researchers have proposed that some ADSCs pre-differentiate into Schwann cells (SCs) whose aim is to have these SCs support intrinsic nerve regeneration [57]. Thus, implantation with ADSC-derived SCs showed improved locomotor function and a reduction in gliosis [60], reduced fibrosis and inflammation [1, 48], and a reduction in neuronal damage [12]. Notably, in these studies, ADSCs appeared to retain their SC function, particularly relating to nerve remyelination [1]. Participation of any of the above mechanisms or others to be discovered in our model is subject of ongoing and future studies. Results from such studies would accelerate bench to bedside translation and streamlining of efficacious treatments of sciatic nerve defects.

## Conclusion

Our present experiments showed that ADSCs may be used as a promising alternative stem cell source for future peripheral nerve injury therapies in lieu of BMSCs. Further studies are necessary to investigate the underlying mechanisms through which ADSCs could support injured nerve regeneration and pave way for prompt bed-side application. Multiple time points would be important to also look at future research, including longer-term follow-up.

## Data Availability

Not applicable

## Abbreviations

ADSCs: Adipose-derived stem cells
MSCs: Mesenchymal stem cells
BMSCs: Bone marrow-derived stem cells
GMI: Gastrocnemius Muscle Index
SFI: Sciatic Functional Index
SCs: Schwann cells
CEANA: Chemically extracted acellular nerve allograft
